# Balancing adaptations and fidelity of implementation strategies to optimize primary healthcare in Ethiopia: Lessons from embedded implementation research

**DOI:** 10.1371/journal.pone.0353519

**Published:** 2026-07-23

**Authors:** Biruk Bogale, Gizachew Tadele Tiruneh, Mesele Damte Argaw, Agumasie Semahegn, Nebreed Fesseha, Chala Tesfaye, Mikiyas Teferi, Hillina Tadesse, Mebrie Belete, Addis Girma, Temesgen Ayehu, Yibeltal Siraneh, Yibeltal Kifle, Bezawit Mesfin Hunegnaw, Dessalew Emaway

**Affiliations:** 1 JSI, Addis Ababa, Ethiopia; 2 University of Global Health Equity, Kigali, Rwanda; 3 Amref Health Africa, Addis Ababa, Ethiopia; 4 MERQ Consultancy PLC, Addis Ababa, Ethiopia; 5 Department of Health Policy and Management, Jimma University, Jimma, Ethiopia; Association for Socially Applicable Research (ASAR), INDIA

## Abstract

**Background:**

Optimizing Ethiopia’s Health Extension Program (HEP) has been critical to enhancing primary healthcare (PHC) service delivery, particularly in remote and underserved settings. Embedded implementation research (EIR) strengthens PHC systems by positioning implementers and local managers at the center of addressing operational bottlenecks, enabling adaptive, context-specific refinement of interventions and strategies while maintaining high fidelity to their core components. This study evaluates the implementation fidelity, adaptation of strategies, and lessons to address challenges in equitable access, quality, and accountability through the “Improve Primary Health Care Service Delivery” project.

**Method:**

A participatory pragmatic EIR approach was applied to co-design and pressure test the implementation strategies to optimize the HEP roadmap (2020–2035). Fourteen woredas from agrarian and pastoral contexts were selected to pressure test HEP optimization strategies. Continuous adaptation tracking and fidelity monitoring were conducted throughout the project implementation period from April 2022 to September 2024. The HEP optimization implementation strategies, adaptations, and modifications were guided by frameworks such as Framework for Reporting Adaptations and Modifications to Evidence-based Implementation Strategies to ensure interventions fit local contexts.

**Results:**

The adaptations of implementation strategies resulted in implementation fidelity, which improved maternal and child health service coverage, strengthened community health program units, enhanced referral systems through Networks of Care (NoCs), and contextualization of service delivery for pastoralist communities. While most strategies were retained and repackaged for scale, others—including performance-based incentives and governance restructuring—were deprioritized because of feasibility, policy, and sustainability considerations. Adaptations were guided through iterative learning and stakeholder engagement and clustered into four broad areas: contextualization of service delivery models, strengthening community engagement, streamlining quality improvement and NoCs approaches, and refining accountability mechanisms. Stakeholder engagement was pivotal in balancing fidelity with contextual adaptations, fostering trust and sustainability. Despite challenges such as resource constraints, sociocultural barriers, and infrastructure limitations, systematic monitoring and iterative learning processes facilitated the refinement of scale-up strategies.

**Conclusions:**

Adaptive implementation science effectively optimizes health programs in complex and dynamic contexts. Participatory co-design, stakeholder engagement, and systematic frameworks facilitated the development and refinement of strategies that improved service quality, access, and accountability in diverse PHC settings.

## Background

Ethiopia’s Primary Health Care (PHC) system—anchored by the Health Extension Program (HEP)—has been instrumental in expanding equitable access to primary health services, particularly maternal and child health care, in rural and underserved areas [1–3]. However, the HEP/PHC program has faced several challenges; poor infrastructure, service fragmentation, poor quality of care, and weak accountability [3–6]. Core components of HEP/PHC, including the delivery of maternal and child health interventions, community engagement practices, and adherence to service delivery protocols, were weakly implemented as intended [2] {Assefa, 2020 #99} limiting the programs potential impact on achieving universal health coverage, and the Sustainable Development Goals [7].

Ethiopia launched the HEP Optimization Roadmap (2020–2035) initiative which outlines the strategic directions and interventions to enhance the HEP’s quality, efficiency, and effectiveness [6–9]. Since 2022, the *Improving Primary Health Care Service Delivery (IPHCSD)* project—implemented by JSI and Amref Health Africa in collaboration with the Ministry of Health (MOH) —has been testing the feasibility of scaling the national strategy by operationalizing selected strategic objectives of the HEP roadmap during the pressure test phase of project implementation (April 2022–March 2024). The project employs an embedded implementation research (EIR) approach and the Networks of Care (NoCs) model to strengthen PHC delivery platforms and improve service integration, coordination, and accountability. As implementation occurs across diverse contexts, project teams have had to navigate the challenge of maintaining intervention integrity while responding to local realities, health system constraints, and emerging implementation needs.

This challenge reflects a longstanding debate within implementation science on balancing fidelity and adaptation. While high fidelity is critical to determine whether observed outcomes can be attributed to the intervention and to preserve its core components, real-world implementation often requires deliberate adaptations to enhance feasibility, acceptability, appropriateness, and responsiveness to context [10,11]. Contemporary perspectives increasingly view fidelity and adaptation as complimentary rather than competing processes: fidelity safeguards the core functions and mechanisms of an intervention, whereas adaptation improves contextual fit and implementation feasibility [11,12]. When systematically planned and guided by clear criteria, adaptations can occur across implementation stages and support intervention sustainability, maturation, and effectiveness without undermining essential functions [13,14]. This balance is particularly important for complex health system interventions that involve multiple interacting components and are implemented within dynamic organizational and community contexts [15,16].

Evidence suggests that both inadequate adaptation and poor implementation fidelity can undermine intervention effectiveness [13,16]. Poorly planned modifications may dilute or alter core intervention functions, whereas rigid adherence to original designs may reduce relevance, acceptability, and feasibility in diverse implementation settings [11,16,17]. Common barriers to fidelity include resource constraints, limited provider capacity, competing priorities, and contextual misalignment, while stakeholder engagement, iterative learning, supportive leadership, and responsiveness to local needs can facilitate effective adaptation and implementation [10,18–20].

Despite growing recognition that both fidelity and adaptation are critical to implementation success, there remains limited empirical evidence on how implementers can systematically balance these competing demands during large-scale health system strengthening initiatives, particularly in low- and middle-income countries [21,22]. Existing literature has largely focused on measuring fidelity or documenting adaptations separately, with less attention given to how adaptation decisions are made, how core intervention functions are preserved, and how fidelity is maintained while responding to dynamic implementation contexts. As a result, important questions remain regarding how implementation teams can adapt interventions without compromising their intended mechanisms of action and how fidelity and adaptation can be assessed together during scale-up

The implementation of Ethiopia’s HEP Optimization Roadmap presents an opportunity to examine these questions in a real-world setting. As roadmap priorities are operationalized through the IPHCSD project, implementers must continually balance adherence to core intervention principles with adaptations required to address local health system realities, resource constraints, and emerging implementation challenges. The aim of this study therefore was to examine how implementation fidelity and contextual adaptation were balanced during implementation of the IPHCSD project in Ethiopia. Specifically, the paper assessed fidelity, documented adaptations, explored how intervention integrity was maintained while adapting to diverse local contexts. The study further identified lessons for balancing fidelity and contextual responsiveness in the scale-up of complex PHC interventions.

## Methods

### Context

The Ethiopian healthcare system is structured in three tiers: primary, secondary, and tertiary. Within the primary framework, the HEP serves as the entry point, linking health posts, health centers, and primary hospitals [4,23]. Launched in 2003, it is the cornerstone of its PHC system, designed to deliver essential health services to rural and underserved communities [24]. By deploying more than 40,000 trained Health Extension Workers (HEWs) to health posts in kebeles (i.e., the lowest administrative unit), the program aimed to achieve universal health coverage for rural households. It has provided 18 packages of preventive, promotive, and basic curative services covering family health, disease prevention, hygiene, and health education [2,25]. Engagement of community structures, such as Women Development Unions (WDUs), has further boosted access to uptake of immunization, maternal and child health services, and communicable disease control [26,27]. To address growing community health service demands and improve reach, Ethiopia introduced the HEP roadmap in 2020, which updated service packages to include non-communicable diseases, mental health, adolescent health, and digital health innovations [7].

### Project description

Since April 2022, JSI and Amref Health Africa (an international non-governmental organizations that support health system strengthening and public health programs), in collaboration with MOH, have been embedding implementation science research [28,29] into the phased implementation of selected strategic objectives of the HEP roadmap, aiming to 1) ensure equitable access to essential health services; 2) improve service quality; and 3) strengthen technical oversight and accountability. The project focuses on improving bidirectional linkages across health facilities to enhance reproductive, maternal, newborn, and child health (RMNCH) outcomes. This was done using the NoCs approach to strengthen the functionality and bidirectional linkages across the PHC delivery platforms [8]—health post to health center to primary hospital—to improve RMNCH) outcomes.

The project has been implemented in three phases to test its feasibility for scale: pressure test, test of scale, and prototyping for scale-up. From April 2022 to March 2024, the project has been pressure-testing operationalization of PHC service delivery packages and modalities in 14 woredas/district (third-level administrative division of Ethiopia, managed by a local government) across nine regions— Agrarian (Amhara, Oromia, Sidama, Central Ethiopia, Southwest Ethiopia, South Ethiopia) and Pastoral (Afar, Somali, Oromia and Gambela). Agrarian woredas generally have relatively better infrastructure, transportation networks, and access to health services. In contrast, pastoral settings are characterized by dispersed populations and high mobility, while limited infrastructure and geographic inaccessibility pose significant challenges to health service delivery and access.

### Study design

This study used an EIR design to examine how HEP optimization interventions were implemented, adapted, and refined during routine delivery. EIR places policymakers, program managers, implementers, and researchers within a continuous learning process to generate actionable evidence while implementation is ongoing. Program implementations were routinely monitored aiming for adaptation and high-fidelity implementation of HEP roadmap interventions to enhance access to and utilization of RMNCH services. Stakeholders including program managers and implementing partners were engaged in refining the implementation strategies and documenting the adaptations guided by the Expert Recommendations for Implementing Change (ERIC) protocol [30]. The study was guided by the premise that effective implementation requires balancing fidelity to core intervention functions with adaptation of strategies forms to local contexts.

### Participatory design of implementation strategies

The intervention under implementation and evaluation was the national HEP Optimization Roadmap, which introduced a package of HEP reforms including restructured HEP service delivery models, health post upgrading, and expanded RMNCH services. The implementation strategies used to operationalize the roadmap were developed through document reviews and stakeholder consultations and included facilitating and advocating for the implementation of the restructured HEP service delivery models, recruiting, training, and deploying VHLs, establishing and strengthening NoCs, implementing quality improvement processes, providing clinical mentorship and supportive supervision, facilitating collaborative learning and adaptive implementation, and strengthening social accountability through community scorecards. These strategies were subsequently mapped to the Effective Practice and Organization of Care (EPOC) group [31], the ERIC taxonomies [30], and HEP Optimization Roadmap strategies [7] (Table 1). A narrative review of the HEP roadmap and other relevant documents [2,5,7,32,33] and stakeholder consultations helped identify multiple implementation challenges, providing insights to guide strategy design. The co-design process identified four major implementation challenges: limited agility of the HEP/PHC system; weak community engagement mechanisms; inequitable access to essential maternal and newborn health (MNH) services, driven by geographic, gender, and sociocultural barriers; suboptimal quality of care; and inadequate accountability and governance within PHC delivery. To address these challenges and pressure-test the feasibility of the HEP roadmap’s strategic objectives, we conducted a participatory process engaging relevant stakeholders to co-design implementation strategies. During the first six months of the project (i.e., April- September 2022), the project team and health system stakeholders collaboratively co-designed service delivery models and interventions tailored to specific contexts. This iterative process resulted in a detailed implementation plan for the investment and initial program theory of change, shaped by intensive consultations.

**Table 1:** Implementation strategies for IPHCSD project, core functions, and adaptable elements, March 2024.

Strategy no.	Strategy	Core function maintained for fidelity	Adaptable forms	EPOC/ERIC classification
1	Facilitate and advocate for the implementation of the restructured HEP service delivery models and health post categorization	Ensure equitable access to essential PHC services through optimized HEP delivery platforms	Categorization criteria, implementation procedures, advocacy approaches, rollout sequencing, contextualization for local settings	Facilitation; Adapt and tailor to context
2	Establish and operationalize Community Health Program Units (CHPUs)	Strengthen multidisciplinary support, coordination, and oversight for community health services	Team composition, outreach modalities, supervision arrangements, service delivery approaches	Facilitation; Service delivery reorganization
3	Train and deploy Village Health Leaders (VHLs)	Strengthen linkage between households, communities, and PHC services	Selection criteria, gender composition, household coverage, job aids and tools	Community engagement; Adapt and tailor to context
4	Strengthen alternative community engagement mechanisms	Improve engagement of underserved population groups and strengthen community participation in PHC	Target populations, engagement platforms, communication channels, delivery approaches	Engage consumers
5	Adapt and operationalize context-specific Mobile Health Services (MHS) models	Improve access to essential health services in hard-to-reach and underserved communities	Site selection, staffing mix, frequency of outreach, management arrangements	Provide community-based services
6	Establish and strengthen NoCs and care coordination mechanisms	Strengthen referral coordination, service integration, and collaboration across PHC facilities and communities	Governance arrangements, engagement of private facilities, learning platforms, operational procedures	Coordination of care; Strengthen referral linkages
7	Implement continuous quality improvement (QI) processes	Promote continuous quality improvement and local problem-solving to improve service delivery	QI tools, coaching approaches, collaborative learning mechanisms, change packages	Audit and feedback; QI
8	Provide clinical mentorship and supportive supervision	Strengthen provider competence and adherence to clinical and service delivery standards	Mentorship modality, frequency, mentor composition, supervision tools	Clinical supervision; Train and educate providers
9	Facilitate collaborative learning and adaptative implementation	Support continuous learning, reflection, and evidence-informed implementation improvement	Learning formats, review mechanisms, documentation processes, feedback platforms	Learning collaboratives
10	Introduce performance-based incentives	Improve provider motivation and performance through incentive mechanisms	Incentive design, performance metrics, verification systems	Organizational change; Financial incentives
11	Strengthen Managerial Accountability Program (MAP)	Strengthen managerial accountability, transparency, and responsiveness within PHC systems	Scoring tools, implementation level, feedback mechanisms, digitalization	Authority and accountability for organizations
12	Revamp community scorecard (CSC) processes	Strengthen social accountability and community feedback on service delivery	Scoring processes, facilitation approaches, community engagement mechanisms, digital tools	Consumer engagement; Community participation
13	Reorganize and implement Primary Health Care Unit (PHCU) governance	Strengthen governance, leadership, and coordination across PHCUs	Governance structures, reporting arrangements, oversight mechanisms	Organizational change; Authority and accountability

HEP: Health Extension Program; PHC: Primary Health Care; ERIC: Expert Recommendations for Implementing Change; EPOC: Effective Practice and Organization of Care; CHPU: Community Health Program Unit; VHL: Village Health Leader; MHS: Mobile Health Services; NoCs: networks of Care; QI: quality improvement; MAPs: managerial accountability for primary health care system; CSC: community scorecard; PHCU: Primary Health Care Unit.

A national co-design workshop was held, bringing together 40 participants, including MOH and regional health bureau (RHB) program managers for HEP/PHC, RMNCH, quality, health system support, and clinical services. During the workshop, the project scope, theory of change, goals, and preliminary strategies were presented. Participants engaged in discussions to identify barriers to HEP Optimization Roadmap implementation. Using the ERIC protocol, stakeholders systematically adapted implementation strategies by contextualizing and validating them and selecting priority strategies to operationalize targeted and innovative PHC service delivery approaches.

**Woreda-level co-creation:** In October–November 2022, the project facilitated woreda-level co-creation and launch workshops in all 14 implementation woredas, engaging 771 participants (360 from agrarian and 411 from pastoral woredas). Participants included representatives from hospitals, health centers, health posts, communities, private facilities, woreda health offices, zonal health departments (ZHD), RHBs, and local universities. Participants collaboratively analyzed assessment findings, identified gaps, and contextualized implementation strategies. Through brainstorming sessions, they prioritized challenges and proposed activities to address them.

### Learning and adaptation approach

JSI, Amref, and learning partner MERQ (Monitoring, Evaluation, Research, and Quality Improvement) conducted several iterative learning processes based on the data from project monitoring and surveys to document the implementations. These iterative learning processes led to adaptations of the project implementation strategies and improved the implementation fidelity. Based on the learnings and adaptations, the project has been scaled up to 30 additional woredas from April 2024 to March 2027.

**Fig 1:**
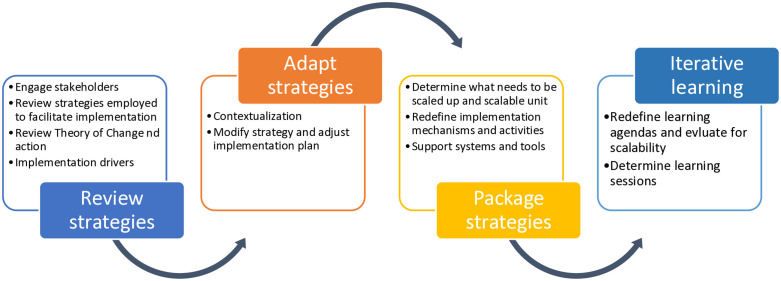
Steps to adapt and package implementation strategies.

The project conducted three annual review meetings for pause and reflection on the implementation strategies with the program leads and implementers, learning partner, and field staff to review strategies for fidelity and identify ongoing challenges and opportunities for adaptations to fit in the context and facilitate scale. In addition, there were bi-weekly meetings and iterative rounds of discussions to identify the program implementation, challenges, and learning throughout the project’s pressure test phase. This began with setting criteria for the implementation strategy’s maturity, including pre-defined timelines, desired outcomes, and geographic coverage. Existing strategies and activities were reviewed and modified as necessary, with targets set for various levels of the health system. As such, strategies and theories of change and action were thoroughly reviewed to ensure fidelity and quality delivery, contextualized, and adapted to local needs to develop a prototype for the test of scale. Implementation drivers crucial for successful scale-up were identified, and strategies were then packaged with scalable units in mind. Implementation mechanisms and activities were redefined, ensuring support systems and tools were in place for a responsive and resilient health system (Fig 1). Accordingly, adaptations were made by balancing adaptation and fidelity. The core elements of the strategies and forms were identified, ensuring their integrity while adapting various aspects. This included modifications and/or adaptations to the intensity, delivery modality, content, setting, personnel, and target population of the strategies and activities. Finally, redefining the learning agendas, and learning sessions were conducted to inform further adaptations and improvements (Table 1).

Additionally, Framework for Reporting Adaptations and Modifications to Evidence-based Implementation Strategies (FRAME-IS) was prospectively used [12], to systematically document adaptations and modifications on the project implementation strategies and interventions. Adaptation components according to context, content, or level of delivery, as well as whether adaptations were planned or unplanned were documented. Adaptations were documented in real time through structured monitoring and learning systems.

### Data and analysis

For this study, project monitoring data, program review findings, and case studies conducted by the project and MERQ were utilized. In addition, service statistics were collected from health facilities covering the period from October 2021 to March 2024. These data were accessed on 20/01/2025.

Quantitative data were summarized using descriptive statistics to assess implementation fidelity and functionality of core components. Fidelity assessments focused on adherence to predefined core functions rather than achievement of service outcomes. Qualitative information from review meetings, supervision reports, workshop minutes, and case studies was analyzed using a framework-based approach informed by FRAME-IS. Adaptations were categorized according to type, rationale, timing, and implementation level. Findings were synthesized to identify patterns in how strategy adaptations preserved, strengthened, or challenged fidelity to core functions.

To enhance analytic rigor, adaptation classifications and interpretations were reviewed jointly by project implementers, researchers, and the learning partner during iterative review meetings. Discrepancies were resolved through consensus and triangulation across data sources.

### Definition and measurement

***Implementation fidelity*** refers to the extent to which strategies and activities are executed as intended, in alignment with the program’s theory of change. To ensure fidelity, we assessed whether strategies were implemented effectively and aligned with project goals, and verified that core components were preserved and delivered as designed, with minimal deviations. It was measured by tracking adherence to critical components and core functions using project monitoring data, covering all implementation phases and the adaptation process. Implementation fidelity is presented for the period from April 2022 to September 2024.

***Adaptation*** refers to the intentional, planned process of modifying activities and strategies to fit the unique context in which they are implemented, while preserving the core elements—the essential components necessary to achieve the intended outcomes. This involves adjusting the forms of the strategy, such as its intensity, delivery modality, content, setting, personnel, or target population. To measure adaptation, we systematically documented and assessed changes made to the strategy’s forms, ensuring that the core elements remained intact. We tracked modifications to aspects like delivery methods, content, intensity, or target population, categorizing these changes as proactive or reactive [12,14]. Additionally, we evaluated the contextual relevance of each adaptation to understand how and why specific adjustments were made to address local needs or challenges. Project monitoring data was used to record and assess these adaptations, ensuring they were deliberate and aligned with the project’s overall goals.

The project’s initial and adapted strategies, implementation outcomes, and results were synthesized from project monitoring data, program reviews, and study reports. Service statistics were extracted from facility records and analyzed to monitor trends in RMNCH outcomes. Program leads and implementers collaborated to critically appraise the implementation strategies during the iterative virtual and in-person meetings.

Table 2 presents the operational definitions, measurement approaches, and indicators used to assess implementation fidelity, adaptation processes, accountability mechanisms, and selected maternal and child health service utilization outcomes during the implementation period.

**Table 2:** Operational definition and measurement of process outcome variables.

Variable	Operational definition and measurement
VHL functionality	VHLs are community volunteers (men and women) with at least Grade 6 education who serve as a linkage between HEWs, community structures, and households. Functionality was assessed based on three criteria: (1) having a community health work plan, (2) submitting performance reports, and (3) participating in review meetings with HEWs.
CHPU functionality	CHPU functionality was assessed using criteria related to team-based community mobilization, home- and outreach-based service delivery, technical support to health posts, and oversight of HEWs.
NoCs functionality	NoCs functionality was measured across multiple domains, including governance and coordination, community engagement, bidirectional referral systems, resource sharing, implementation of clinical standards, QI, capacity strengthening, learning and collaboration, gender integration, respectful people-centered care, private-sector engagement, and bidirectional accountability.
NoCs maturity	NoCs maturity was assessed using four domains: purposeful arrangement, operational standards, quality and accountability, and learning and adaptation. Scores ranged from 1 (emerging) to 4 (fully mature).
Managerial Accountability for Primary Health Care System (MAPs) score	MAP implementation fidelity was assessed across five domains: transparency, inclusive decision-making, performance management, stakeholder participation, and managerial responsiveness within PHC systems.
CSC score	CSC performance was measured using scores assigned by community representatives and client councils on motivation, provider competence and compassionate care, waiting time, availability of diagnostics and medicines, facility infrastructure, ambulance services, and facility cleanliness.
Contraceptive acceptance rate (CAR)	Percentage of women who adopted or continued using modern contraceptive methods. Calculated as the number of women receiving family planning services divided by the expected number of non-pregnant women in the catchment population, multiplied by 100. The expected number of non-pregnant women was estimated as 20% of the catchment population.
ANC 4 + contacts	Percentage of pregnant women who attended at least four ANC contacts. Calculated as the number of women completing four or more ANC visits divided by the expected number of pregnancies, multiplied by 100.
ANC 8 + contacts	Percentage of pregnant women who attended at least eight ANC contacts. Calculated as the number of women completing eight or more ANC visits divided by the expected number of pregnancies, multiplied by 100.
Skilled birth attendance (SBA)	Percentage of births attended by a skilled health professional. Calculated as the number of skilled birth attendant-assisted deliveries divided by the expected number of pregnancies, multiplied by 100.

Note: For ANC4 + , ANC8 + , and SBA indicators, the expected number of pregnancies was estimated as 3.4% of the catchment population. For the CAR, the expected number of non-pregnant women was estimated as 20% of the catchment population. Service utilization indicators are presented as descriptive measures of implementation context and are not used for causal attribution of project effects.

VHL: Village Health Leader; HEW: Health Extension Worker; CHPU: Community Health Program Unit; NoCs: networks of Care; QI: quality improvement; MAPs: Managerial Accountability for Primary Health Care System; PHC: Primary Health Care; CSC: community scorecard; CAR: contraceptive acceptance rate; ANC: antenatal care; SBA: skilled birth attendance.

### Reflexivity statement

The research team consisted of implementing organizations (JSI and Amref Health Africa), a learning partner (MERQ), and collaboration with the MOH and subnational health authorities. The team therefore held dual roles in implementation support and embedded evaluation. To mitigate potential bias arising from this embedded position, multiple strategies were applied, including triangulation of data sources, use of standardized analytic frameworks (ERIC and FRAME-IS), and structured documentation of decision-making processes. Reflexive notes were maintained during implementation learning cycles to document contextual influences on adaptation decisions.

The study is reported in accordance with the Standards for Reporting Implementation Studies (StaRI), ensuring transparency in reporting implementation design, processes, and outcomes.

### Ethical approval

We used data captured through routine project monitoring, as well as minutes from meetings and workshops conducted during project implementation. Routine monitoring data collected between April 2022 and September 2024 were analyzed. The project received ethical approval for the implementation research from the Ethiopian Public Health Association Institutional Review Board (IRB) on February 19, 2024 (Reference No. EPHA/OG/159/24). The IRB waived the requirement for client informed consent; however, we obtained consent from health facility directors and woreda health offices. Retrospective service statistics were obtained from medical records as monthly aggregated data, which were fully anonymized prior to access. Similarly, all information gathered was de-identified before analysis. Throughout all stages of the study, strict confidentiality was maintained, and the research was conducted in full compliance with the ethical principles. We confirm that the study was conducted in full compliance with the principles of the Declaration of Helsinki.

## Results

### Fidelity of implementation during pressure testing

Across the pressure-testing phase, fidelity to core strategies was largely maintained across both agrarian and pastoralist settings despite substantial contextual adaptations in implementation forms. The implementation fidelity of these strategies is presented below.

### Service delivery packages for HEP operationalized

The project operationalized the HEP service delivery modalities across 273 health posts, restructured into three categories: 1) 15 comprehensive health posts (CHPs), 2) 207 basic health posts, and 3) 51 merged into health centers and primary hospitals). This contributed to ensuring equitable access to essential health services in 14 intervention woredas.

The project facilitated the establishment of CHPU at 66 health centers and 6 primary hospitals, where multidisciplinary teams provide healthcare services and backstopping support for the catchment health posts. Lessons learned from the CHPU implementation enhanced both static and team-based outreach services, contributing significantly to the operationalization and implementation of the HEP optimization roadmap; the program implementers witnessed that the establishment of CHPU enhanced multidisciplinary healthcare provision, enhanced collaboration, and teamwork among health workers; improved communication, coordination, performance of healthcare, and improved outreach health service delivery [8,34].

The functionality score of CHPU from June 2023 to March 2024 was measured against criteria developed on its facilitation of team-based community mobilization, home-based and outreach-based service delivery, technical support, and oversight of HEWs. The scores have improved from 72% to 79% in agrarian woredas and from 37% to 62% in pastoral woredas (Table 3).

**Table 3:** Implementation fidelity of strategies, April 2022-March 2024.

Strategies	Implementation fidelity
Facilitate and advocate for the implementation of the redesigned HEP service packages and delivery modalities	• HEP service delivery modalities operationalized across 273 HPs: 51 merged (35 in agrarian and 16 in pastoral), 207 basic, and 15 CHPs • 921 (agrarian: 697 and pastoral: 224) HEWs and other providers trained on revised HEP • Advocacy for HEP optimization (to mobilize resources and attention) conducted at all levels
Establish and operationalize CHPU at health centers and primary hospitals	• 66 health centers and 6 primary hospitals established CHPUs (agrarian: 43 health centers and 4 primary hospitals and pastoral: 23 health centers and 2 primary hospitals) • CHPU enhanced both static and team-based outreach services, contributing significantly to the operationalization and implementation of the HEP optimization roadmap; the program implementers witnessed that the establishment of CHPU enhanced multidisciplinary healthcare provision, enhanced collaboration, and teamwork among health workers; improved communication, coordination, performance of healthcare, and improved outreach health service delivery.
Contextualize service delivery modalities contextualized for pastoralist settings	• Woreda-led MHS implemented across five woredas (covering 17 PHCUs and 59 hard-to-reach sites), conducting 22 rounds and 196 sessions and reaching 40,495 individuals (17,030 males and 23,465 females) with over half being MNH service beneficiaries.
Strengthen community engagement strategies	• 3,790 VHLs (2,484 in agrarian and 1,306 in pastoral) recruited, trained, and deployed at all intervention woredas • VHL strategy contextualized to pastoral regions;(Makafta in Afar, Reer in Somali, Rera in Borena, Eagn in Dasenech) • Implementation guides and monitoring tools were developed • Non-momentary incentive schemes introduced for community health workers • The majority of the VHLs were active and collaborate with WDU for community health activities • Program showed VHLs reached 84% of households; identified 46% of pregnant women, 21% of zero-dose children; linked 30,000 + women to family planning
Establish and strengthen NoCs across PHC facilities	• 66 PHCUs (agrarian: 43 PHCUs and pastoral: 23 PHCUs), 6 Primary Hospitals, and 53 private PHC facilities established NoCs in 14 woredas (8)
	• Functionality of NoCs facilities strengthened • Facilitated collaborative learning amongst facilities in the network • Overall, we learned that the implementation of NoCs has notably fostered improved trust and communication among team members, improved continuity of care, strengthened the referral system, facilitated resource sharing and operational efficiency, facilitated the functional establishment of new CHPs, enhanced competency levels among healthcare providers and ensured better adherence to clinical standards and tools, and engendered greater community ownership and support. • Perinatal mortality rate decreased from an average of 31.3 per 1,000 births to 21.0 per 1,000 births during March 2022-February 2023 to 4.4 during March 2023-February 2024 (8)
Implement internal continuous quality improvement processes	• HEP quality service delivery standards developed • Catchment-based mentorship guidelines and tools for HEP developed • clinical audits introduced • 418 providers trained in QI (267 in agrarian and 151 in pastoral) • 80 QI projects have been initiated, with 21 already completed; 66 health centers (43 in agrarian and 23 in pastoral), 14 hospitals, and 26 health posts in agrarian woreda implementing QI projects • Established community QI teams and activated facility-level QI teams
Implement performance-based incentive schemes	• Dropped
Streamline and strengthen MAPs	• 42 managers trained on MAPs and CSC. • Health system responsiveness scores showed promising trends, whereas the lowest scores were observed in stakeholder engagement in the scoring process.
Revamp community scorecard	• The mean CSC score increased from 74% to 80% between July-September 2023 and January-March 2024 in agrarian settings, while in pastoral settings it stalled at 75% during October-December 2023. • Respectful care, facility cleanliness, and waiting time performed better, whereas the availability and management of ambulances, facility infrastructure, and drugs and supplies received the lowest scores.
Implement administrative and clinical primary care reforms	• 66 health centers and 6 primary hospitals implementing primary care reform guidelines • Health post reform implementation guide implementation improved the performance of the health posts in providing community health services over time
Reorganize and implement the governance structure of PHCU	• Dropped

HEP: Health Extension Program; CHPU: Community Health Program Unit; NoCs: networks of Care; QI: quality improvement; MAPs: Managerial Accountability for Primary Health Care System; CSC: community scorecard; PHCU: Primary Health Care Unit.

Contextualized health service delivery method to hard-to-reach areas is one of the service provision modalities integrated with the routine health service delivery. Woreda-led MHS have been designed and provided by multidisciplinary teams, covering 17 PHCUs and 59 hard-to-reach sites, and offering services to 40,495 vulnerable community members (17,030 males, 23,465 females), with over half being MNH clients (Table 3).

### Community engagement strategies strengthened

The project trained and deployed 3,790 VHLs (2,484 in agrarian and 1,306 in pastoral) at all intervention woredas. VHL strategy contextualized to pastoral regions: (Makafta in Afar, Reer in Somali, Rera in Borena, Eagn in Dasenech). Between July 2023 to March 2024, the functionality of VHL has improved from 75.5% to 90% in agrarian settings, and more than 80% of VHLs collaborated with WDUs during their community activities. Moreover, non-monetary incentives were designed and implemented for community health workers as a motivation scheme.

### NoCs for PHC facilities established

**Fig 2:**
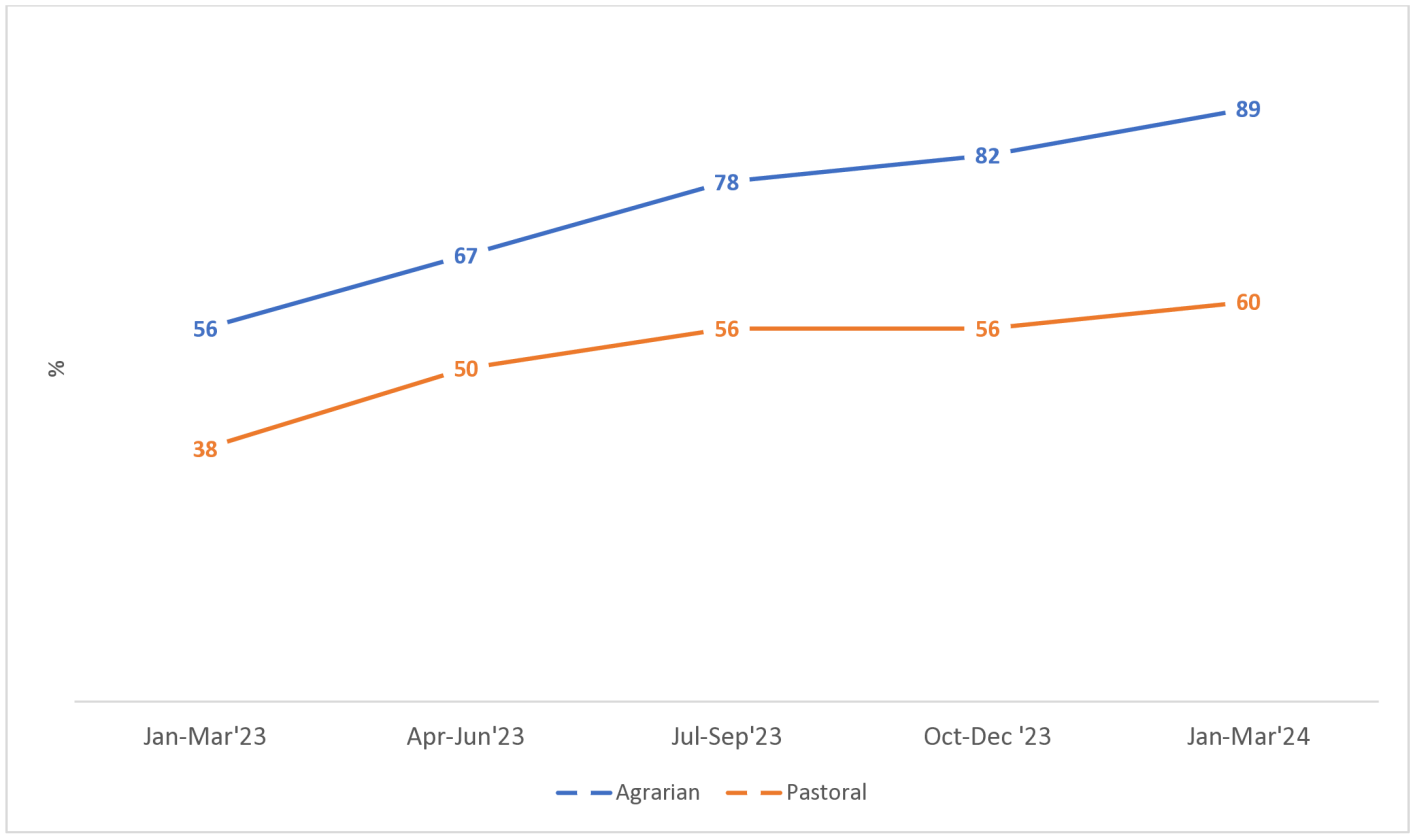
Trends in NoCs functionality score in agrarian and pastoral settings, January 2023 to January 2024.

A NoCs for PHC facilities was established to enhance coordination, referral systems, and capacity building across facilities (health posts, health centers, private health facilities, primary hospitals) and communities. Additionally, the project implemented a hub and spoke model as part of the NoCs implementation, particularly focusing on pastoral regions. This model notably fostered trust and communication among team members, improved continuity of care, strengthened the referral system, and facilitated resource sharing, operational efficiency, and technical competency of providers [8]. The NoCs functionality score has improved from 56% to 89% in agrarian settings from January 2023 to March 2024, while it has improved from 38% to 60% in pastoral settings (Fig 2).

### PHC service delivery processes optimized

The QI initiative, which involved the development of 80 QI projects at health facilities, organizing 21 learning collaboratives at the woreda level, and the establishment of a combined facility-community quality improvement process in the South Bench woreda. As a result, the project has documented improvements in the quality of care across all intervention woredas and a notable reduction in perinatal mortality rates (decreased by 34%, from 31.3 to 20.1 per 1,000 births) in South Bench (Table 3). Furthermore, the project included the implementation of catchment-based clinical mentorship through training and facilitating knowledge and skill development activities for healthcare professionals.

### Bi-directional accountability for PHC across the health system implemented

To enhance bi-directional and social accountability across PHC, the project implemented a CSC and MAPs collaboration with various offices within the MOH. Following training, health facilities facilitated quarterly scoring for both CSC and MAPs, where community members give scores for the service delivery at the health facilities, and the healthcare providers give scores for their supervisors. The mean CSC score increased from 74% to 80% between July-September 2023 and January-March 2024 in agrarian settings, while in pastoral settings it stalled at 75% during October-December 2023 (Table 3). These efforts have significantly enhanced the health system’s responsiveness to community demands, marking a notable advancement in accountability and governance within the PHC system.

### Types and patterns of adaptations

#### Adaptations across implementation phases.

While fidelity to core functions was maintained, several interventions were modified, refined, deprioritized, or discontinued to improve feasibility, contextual fit, and scalability. For example, performance-based incentives and PHCU governance restructuring were deprioritized [35]. because of challenges related to performance measurement and verification, as well as limited evidence on their effectiveness and policy limitations.

**Fig 3:**
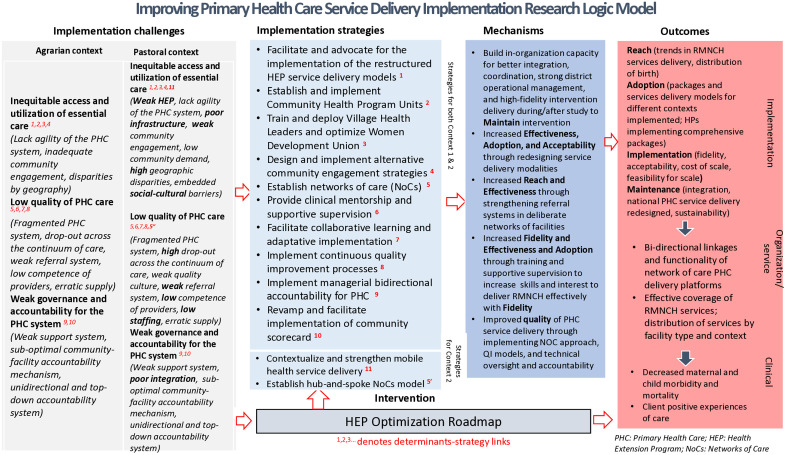
Revised project theory of change.

During the transition from the pressure test phase to the scale implementation phase, iterative consultations with MOH, donor, program managers, and implementing partners were used to review implementation experiences and assess the balance between fidelity and adaptation. Consequently, the project theory of change was refined (Fig 3). Analysis of the adaptation process identified four major adaptation patterns that informed the design and packaging of interventions for the test-of-scale phase (Table 4).

**Table 4:** Strategies adapted and packed for the test of scale, March 2024.

Strategies	Implementation challenges during pressure-testing	Adaptations	Repacked for the test of scale
Facilitate and advocate for the implementation of restructured HEP service delivery models	• Unclear health post categorization criteria • Inadequate infrastructure and human resources for CHPs • Inconsistent understanding of the reform package across regions	• RHB/ZHD level advocacy • Contextualize health post categorization criteria (enhance community engagement in the process • Advocate for phase-based introduction/construction of CHPs • Revisit health post reform implementation guide and translation into the local language	• Twinning (adjacent pressure test woreda was assigned to coach and mentor new test of scale woreda) for experience sharing • Develop advocacy strategy (and tools) and facilitate policy dialogues • Optimize basic health posts (intensified outreach service, community engagement, advocate resources, and mentorship, ensure HEWs spend the right time at health post, and open house sessions) • Strengthen health post reform implementation guide • Introduce integrated clinical guidelines for the CHP
Strengthen community engagement	• Inconsistent functionality of VHLs and WDUs • Low motivation of community volunteers	• Further contextualization selection criteria: gender mix, number of households per VHL (Reer, Rera, Makafta, Eagn) • Strengthen working relationships with WDUs and other community health workers • Contextualize WDUs (‘Umergargar’ and ‘Haadha Sinqee’, Abbaa Feyyaa)	• Scale VHL strategy • Design and test youth and men engagement • Conduct functionality assessment • Introduce non-financial motivation mechanisms • Optimize WDUs (manageable size and contextualization)
Contextualize and strengthen mobile health service (MHS) delivery	• Limited reach to the most remote populations • Sustainability concerns due to high operational costs • Weak integration with local financing mechanisms	• Revisit the MHS site selection criteria and prioritize hard-to-reach sites • Advocate for the integration of MHS with community-based health insurance	• Institutionalize MHS • Introducing innovative financing for MHS • Facilitate the provision of basic health services for selected hard-to-reach areas.
Establish and operationalize NoCs across PHC facilities.	• Weak functionality of some NoCs • Unclear objectives and accountability mechanisms • Limited engagement of private facilities • Duplication with existing review platforms	• Revisit the NoCs guide (set precise SMART objectives, align with EPAQ and PHC SBFR, • Streamline NoCs to optimize HEP. • Optimize private facilities engagement (redefine the role of private facilities in capacity building, data exchange, and performance review) • integrate NoCs collaborative learnings into regular PHCU-level meetings, woreda-level quarterly performance review	• Facilitate implementation of NoCs and hub and spoke models • Integrate NoCs approaches into the woreda health system workstream • Implement the hub and spoke model in both pastoralist and agrarian areas
Implement continous quality improvement processes	• Limited QI capacity at facility and community levels • Inconsistent use of data for decision-making • Weak coaching and collaborative learning mechanisms	• Revisit the combined community-facility QI guide • Intensify collaborative learning and enhance data use	• Package QI change ideas and scale • Strengthen QI coaching • Introduce automated Excel tracking
Provide catchment-based clinical mentorship	• Irregular mentorship visits; limited availability of qualified mentors; lack of standardized mentorship tools; • High geographic dispersion of facilities	• Revise the health post mentorship tool and the modality • Document learnings and prototype it for the test of scale	• Phase-based onsite and remote mentoring • Establish a pool of mentors for health post mentoring • Synthesis of learnings and position for the test of scale
Implement performance-based incentives	• Concerns regarding sustainability and financing • Lack of alignment with national policies • Emergence of performance-based financing as a higher policy priority	• Deprioritized	• Deprioritized • Performance-based financing is the focus for MOH and RHBs
Revamp and implement community scorecards and MAPs	• Limited use of accountability data for action • Cumbersome paper-based scoring systems • Inconsistent completion of the feedback and response cycle	• Reorient CSC and MAPs to optimize HEP • Digitize the MAPs scoring tool	• Facilitate scale implementation • Strengthen the full cycle of CSC and MAPs • Pilot MAPs tools at the zone health department level
Reorganize the governance structure of PHCU	• Limited policy and administrative support for restructuring PHCU governance • Concerns about feasibility within existing health system arrangements	• Deprioritized	• Deprioritized • Lack of policy support for the reorganization of the PHC governance structure

HEP: Health Extension Program; RHB: regional health bureau; ZHD: zonal health department; WDU: Women Development Union; NoCs: Networks of Care; QI: quality improvement; MAPs: Managerial Accountability for Primary Health Care System; CSC: community scorecard; PHCU: Primary Health Care Unit.

**Theme 1: Contextualizing and streamlining strategies to operationalize service delivery packages and models:** To improve access to and use of health services, the project made a number of strategic adjustments during the pressure test phase. Therefore, adaptation was made on the categorization and re-categorization based on criteria and considering other topographic barriers. Technical support and training were given to facilitate the categorization and re-categorization of the HEP, which in turn improved health service access in the rural community. Effective stakeholder engagement—including communities, local leadership, and healthcare providers—was crucial for health post categorization, improved VHL acceptance, and maternal and child health service uptake.

An open house session strategy was adopted to improve health literacy, where the CHPs and basic health posts organize an exhibition of healthcare services provided at their facility for their nearby communities. This approach continued during the test of scale with the introduction of integrated clinical guidelines for CHPs. Moreover, to optimize the efficiency of the woreda-led MHS approach and sustainability, the professional mix was contextualized to include social workers and volunteers and shift the management from the woreda health office to health centers. Institutionalizing the MHS and introducing innovative financing schemes were re-packaged for the test of scale.

**Theme 2: Adapting community engagement strategies:** To strengthen community engagement, the VHL strategy was adapted to ensure balanced gender-mixed teams, adjusted household numbers, and provided refresher training and tools (cue cards and checklists) for better performance. Scaling the VHL strategy, testing the youth and men engagement, and optimizing the WDU were a repackaged strategy for the test of scale phase. Despite these modifications, the core function of linking communities with the PHC system remained unchanged.

**Theme 3: Streamlining QI and network functions:** Despite the successful implementation of NoCs and QI strategies, the implementation has faced several challenges. Coordination among facilities and communities has been difficult due to the large number of Plan-Do-Study-Act (PDSA) cycles and QI teams. In addition, the broad goals of the NoCs lacked clarity and alignment of objectives across all levels, along with limited engagement with private healthcare facilities, inconsistent use of QI tools, inadequate leadership skills, and transportation difficulties impacted the NoCs and QI efforts.

To address these challenges and streamline NoCs implementation for clarity and effectiveness, the project adapted precise objectives for NoCs and aligned interventions with essential quality and service standards. This includes revising the NoCs guidelines to detail specific activities within each NoCs domain. The roles of private facilities were redefined to incorporate them into capacity building, data exchange, emergency response, and joint performance reviews. Additionally, the project integrated NoCs collaborative learning and NoCs steering committee meetings into regular PHCU-level meetings, woreda-level quarterly performance review meetings, and catchment-based mentorship strategies. Quality improvement is emphasized by introducing change bundles, QI coaching, and establishing a dedicated QI database. For the test of scale phase, integration of NoCs and hub and spoke models into the Woreda Health workstream, QI scale-up and coaching, and introduction automated Excel-based tracking tool were re-packaged for the test of scale phase. These modifications improved operational feasibility while maintaining the core functions of referral coordination, collaborative learning, and continuous QI.

**Theme 4: Adapting accountability mechanisms:** To consolidate the gains, the project has significantly contributed to the revision of both CSC and MAPs guidelines, led by the MOH. Piloting the MAPs implementation at the Zonal Health Department, facilitating full cycle implementation, and digitizing the tools were re-packaged for the test of scale.

### Adaptation–fidelity balance and adaptation governance

Despite extensive contextual modifications, fidelity to core functions of the strategies was largely maintained throughout implementation. Core functions preserved included community linkage through VHLs, service delivery models, referral coordination through NoCs, and continuous QI processes (Table 1). Fidelity was supported through structured monitoring systems, supportive supervision, and collaborative learning platforms.

Adaptations were governed through iterative decision-making processes involving the MOH, RHBs and ZHDs, implementing partners, and facility-level stakeholders. Regular review meetings, supervision visits, and learning sessions created continuous feedback loops that informed real-time adjustments. The principal implementation finding was that fidelity and adaptation functioned as complementary rather than competing processes. Most adaptations modified forms—including delivery modalities, implementation arrangements, staffing configurations, and engagement approaches—while preserving intended intervention functions. Several interventions were refined and repackaged for scale-up, including NoCs, VHLs, MHS, CSC, and the MAPs.

### Repackaging strategies for scale

Based on lessons from pressure testing, implementation strategies were refined and repackaged for scale-up. Key refinements included simplification of NoCs objectives, strengthening of CHPU roles, institutionalization of mobile health services, scaling of VHL strategies, and integration of accountability mechanisms into routine health system platforms. Performance-based incentives and governance restructuring of PHCUs were de-prioritized.

To facilitate the implementation and scale-up of repackaged strategies, the project strengthened support systems, tools, and performance management mechanisms. Nationally adopted strategies such as the MAPs and CSC were refined and scaled up, while additional strategies were packaged and positioned for broader implementation. Experience-sharing and learning visits were organized in collaboration with regional and zonal health bureaus to the test-of-scale woredas, enabling peer learning and promoting ownership among local implementers. These engagements provided platforms for contextualizing implementation approaches and refining delivery modalities based on practical lessons.

Performance management and supportive supervision systems were adapted to sustain implementation fidelity and learning. The project conducted quarterly supportive supervision using structured tools and complemented this with biweekly and monthly internal performance reviews. Biannual collaborative performance reviews and learning meetings provided opportunities to identify challenges and enhance strategy execution. During the test-of-scale phase, the health system has taken the lead in conducting quarterly supervision, while the project provides biannual joint supervision with a simplified checklist. Annual regional review meetings are also planned to recognize best-performing woredas, facilities, and community volunteers. To further promote cross-learning, the project is establishing a Telegram-based virtual platform to enable continuous, cluster-based learning among intervention woredas.

The implementation arrangement for the scale-up follows a structured, partnership-driven approach emphasizing cluster-based co-creation, sub-granting, twinning, integration with partners, and communication and advocacy. Cluster-based co-creation workshops bring together adjacent and pressure-tested woredas to contextualize strategies, conduct bottleneck analyses, and jointly plan interventions. Sub-granting continues through five local implementing partners to strengthen woreda- and facility-level implementation, complemented by Amref’s direct facilitation in 21 woredas. Through the twinning approach, pressure-tested woredas mentor test-of-scale woredas via joint reviews and learning visits.

### Improved access to and use of maternal and child health services

**Fig 4:**
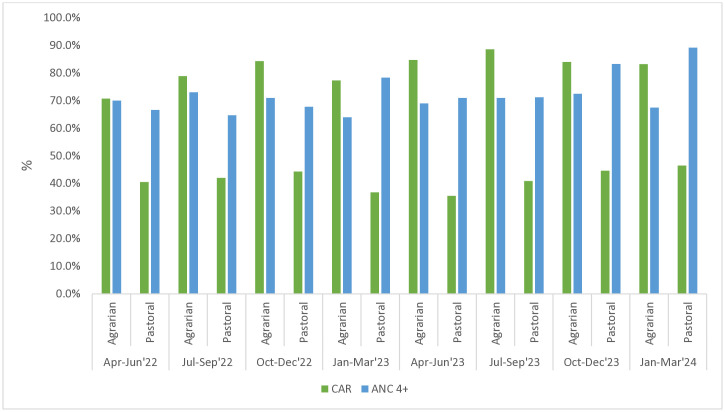
Trends in Coverage of CAR and ANC 4 + visits, October 2021 to March 2024.

The service statistics data showed improved access to and delivery of maternal and child health services in remote communities. In agrarian settings, CAR increased from 71% to 83% following the intervention, while in pastoral areas it rose from 40.5% to 46.5% (Fig 4).

**Fig 5:**
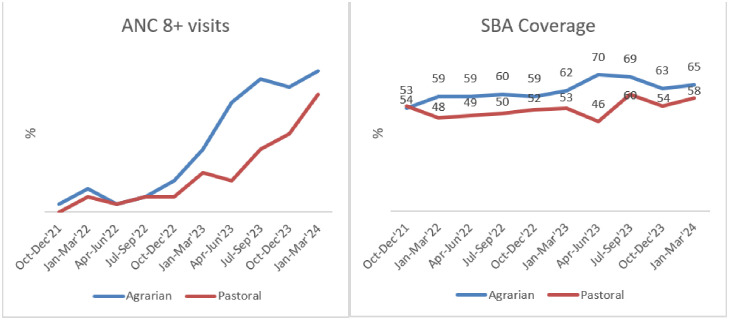
Trends in coverage of ANC 8 + visits and SBA, October 2021 to March 2024.

Skilled birth attendance (SBA) showed a notable improvement, rising from 58% to 65% in agrarian areas and from 50% to 54% in pastoral areas. Encouragingly, ANC 8 + visits also demonstrated progress, increasing from 2% to 18% in agrarian settings and from 1% to 15% in pastoral settings (Fig 5).

## Discussion

This embedded implementation research demonstrated that fidelity and adaptation functioned as complementary rather than competing processes, showing that preserving core functions while adapting implementation approaches can facilitate the scale-up of complex PHC interventions in diverse and resource-constrained settings. Across agrarian and pastoralist contexts, core intervention functions were maintained while intervention forms were adapted to enhance contextual fit, feasibility, acceptability, and scalability. Adaptations were guided through iterative learning and stakeholder engagement and clustered into four broad areas: contextualization of service delivery models, strengthening community engagement, streamlining QI and NoCs approaches, and refining accountability mechanisms. While most interventions were retained and repackaged for scale, others—including performance-based incentives and PHCU governance restructuring—were deprioritized because of feasibility, policy, and sustainability considerations.

The project operationalized the HEP roadmap and substantially strengthened HEP/PHC by improving supervision, referral systems, teamwork, and motivation among HEWs, while increasing community satisfaction, service uptake, and RMNCH coverage. Adaptations and scaling of HEP strategies enhanced accessibility, acceptability, and continuity of care, particularly benefiting MNH. Ensuring fidelity is critical for the successful implementation of evidence-based programs in a complex health system environment [13,36]. Through tailored and context-specific modifications and adaptations fidelity of the intervention could be maximized [37]. The project achieved notable fidelity in implementing key strategies, such as community engagement, QI initiatives, and governance strengthening. This alignment with the program’s theory of change ensured that the interventions retained their intended purpose and functionality, thereby enhancing their effectiveness. Routine monitoring, stakeholder consultations, and participatory reviews, including bi-weekly and annual reflection meetings, facilitated continuous oversight and adherence to the intervention’s critical components.

These findings highlight that adaptive, context-specific strategies can drive meaningful improvements in HEP/PHC performance, but sustained success and broader scale-up require strengthened governance, capacity building, and coordinated oversight. Participatory co-creations and consultation processes with stakeholders were transformative approaches in public health programs, ensuring that interventions are inclusive, contextually appropriate, and effective [38–40]. Our findings suggest that the use of co-creation workshops and continuous stakeholder engagement facilitated the balance between fidelity and adaptation during implementation. Consistent with implementation science literature, involving community members, health workers, managers, and policymakers throughout the design and implementation process improved contextual fit, fostered local ownership, and enabled the intervention to respond to emerging implementation challenges. Previous studies have shown that co-creation and stakeholder consultation support the adaptation of interventions to local contexts while preserving core functions, thereby enhancing acceptability, feasibility, and implementation fidelity. Moreover, iterative engagement with frontline implementers and communities can strengthen commitment to intervention delivery and facilitate integration into existing health system structures [41–43]. Adaptation played a pivotal role in overcoming contextual challenges and enhancing the fit of interventions [10,11]. This finding is consistent with implementation science literature, which recognizes adaptation as a deliberate process of modifying intervention delivery to improve contextual fit, feasibility, and effectiveness while preserving core intervention functions [44,45]. Studies have shown that interventions implemented in new settings often require adaptations to accommodate contextual realities, stakeholder needs, and health system capacities, and that successful adaptations are typically informed by stakeholder engagement and iterative learning processes [46]. Furthermore, contemporary implementation frameworks emphasize that fidelity should focus on preserving core functions and mechanisms of action while allowing flexibility in implementation strategies and delivery approaches [16,47]. In our study, tailored approaches for pastoralist communities, including restructuring HEP implementation modalities, introducing MHS, and strengthening community engagement through open house sessions, improved access, awareness, and service utilization. These findings support growing evidence that balancing fidelity with contextually appropriate adaptation enhances implementation effectiveness and facilitates the scale-up of complex health system interventions across diverse settings.

The success of adaptations underscores the importance of using systematic frameworks, such as the FRAME-IS [12], to guide modifications while preserving the core elements of interventions. These findings align with existing literature emphasizing the role of adaptive management in scaling impactful health interventions in dynamic contexts. [11,13]. Moreover, meaningful engagements of stakeholders in the adaptation of program implementation strategies were effective and drew lessons for program managers and policymakers.

This study reinforces the ongoing debate around fidelity and adaptation. While fidelity ensures the integrity of evidence-based interventions, adaptation addresses the realities of implementation settings. A key lesson from this research is the value of stakeholder engagement in achieving this balance. Participatory approaches, such as the co-creation of strategies and iterative feedback loops, proved instrumental in refining interventions without compromising their effectiveness. Stakeholder engagement strategies, including establishing mutual trust, communicating clearly, and asking for input, facilitate effective adoption, implementation, and sustainability of evidence-based interventions [48,49]

However, the study also highlighted challenges to fidelity, including limited infrastructure, resource constraints, and sociocultural barriers, particularly in pastoralist and remote regions. These challenges occasionally necessitated deviations, such as deprioritizing healthcare worker incentive designs due to implementation complexities. These findings demonstrate the delicate balance required between maintaining fidelity and allowing necessary flexibility for context-specific adaptations.

Furthermore, the study identified the need for adaptive learning systems to monitor and respond to emerging challenges. By integrating bi-weekly reviews, collaborative learning platforms, and stakeholder consultations, the project demonstrated the feasibility of real-time adaptations while maintaining overall program coherence. These iterative learning and review processes helped program managers and implementers in responding to contextual challenges, including conflict, epidemics, and flooding. In addition, it facilitated the refinement of learning questions and re-packaged the intervention strategies for scale-up. Moreover, systematic documentation of these adaptations increases our ability to describe the adaptation process and its impact and replicate them in other settings [12,18,50].

Our study’s strengths lie in its comprehensive and systematic approach to implementation research, integrating robust frameworks like FRAME-IS and ERIC to ensure credible and replicable findings. By focusing on Ethiopia’s diverse healthcare contexts, the study offers context-specific insights while effectively balancing fidelity to intervention strategies and necessary adaptations for local relevance. Moreover, the participatory co-design approach, involving a wide range of stakeholders, enhanced ownership and sustainability, while real-time monitoring and learning mechanisms enabled continuous refinement of strategies.

While this study provides valuable insights, it is not without limitations. The pressure-testing phase was limited to 14 woredas, which may not fully capture the diversity of Ethiopia’s PHC landscape. Additionally, the reliance on routine monitoring data and qualitative insights limits the generalizability of findings. Future research should explore the long-term impact of adaptations on health outcomes and the cost-effectiveness of scaling these strategies.

## Conclusion

This study highlights the critical interplay between implementation fidelity and adaptation in optimizing HEP. The stakeholder engagement, iterative co-design process, well-aligned collaborative partnership between implementers and research team, and contextualization enhanced the implementation fidelity and ease of adaptation process. By balancing these elements, the project achieved significant progress in improving access, quality, and accountability in PHC delivery. These lessons provide a valuable blueprint for policymakers and practitioners seeking to scale impactful health interventions in complex and dynamic contexts.

## Supporting information

S1 File Service statistics dataset used for analysis. (XLSX)

## Abbreviations

ANC: antenatal care
CAR: contraceptive acceptance rate
CHP: comprehensive health post
CHPU: community health program unit
CSC: community scorecard
EIR: embedded implementation research
ERIC: expert recommendations for implementing change
FRAME-IS: framework for documenting modifications to implementation strategies
HEP: health extension program
HEW: health extension worker
IPHCSD: improving primary health care service delivery project
MAPs: managerial accountability for primary health care system
MHS: mobile health service
MNH: maternal and newborn health
MOH: Ministry of Health
NoCs: networks of care
PHC: primary health care
PHCU: primary health care unit
QI: quality improvement
RHB: Regional Health Bureau
RMNCH: reproductive, maternal, newborn, and child health
SBA: skilled birth attendance
VHL: village health leader
WDU: women development union
ZHD: Zonal Health Department
